# Late stage diagnosis of mucinous adenocarcinoma of the appendix: a case report of an unusual tumor with a rare presentation

**DOI:** 10.1186/s12876-020-01378-w

**Published:** 2020-08-21

**Authors:** Katerina Roma, Mark Baldwin, Daniel Sedmak, Matthew Silva, William Stellar, Gina Many

**Affiliations:** 1 Pacific Northwest University of Health Sciences, Yakima, WA 98901 USA; 2 Veteran’s Hospital, Roseburg, OR 97471 USA; 3 Community Health of Central Washington, Yakima, WA 98901 USA; 4 Cascade Surgical Partners, Yakima, WA 98901 USA; 5 The Ohio State University, Columbus, OH 43210 USA

**Keywords:** Case report, Appendiceal cancer, Inguinal pain, Inguinal ulcer, Scrotal abscess, Hematuria, Bladder mass

## Abstract

**Background:**

The incidence of mucinous appendiceal adenocarcinomas (MAA) has increased over the past three decades. Advanced stage tumor diagnosis is likely attributable to non-specific findings. Here we describe advanced stage appendiceal MAA presenting as inguinal ulcers, scrotal abscesses, and other nonspecific symptoms. To our knowledge, this is the first report of MAA presenting as inguinal pain with inflamed phlegmonous tissue and scrotal abscess.

**Case presentation:**

A 67-year-old male presented to a rural facility complaining of weight-loss, fatigue, hematuria, dysuria, painful right inguinal ulceration, and right scrotal abscess drainage. Computed tomography of the abdomen and pelvis revealed a distended appendix (> 1.3 cm) and a fistula between the appendix, urinary bladder, right scrotum, and right groin. Laparoscopic appendectomy was performed and diagnosed as MAA. After a right hemicolectomy, the MAA was staged as pT3b pN0 M0 G2.

**Conclusion:**

This case highlights a unique presentation of late stage appendiceal MAA. Due to the increased incidence of appendiceal MAAs, reports of unique clinical features are needed to facilitate early diagnosis and intervention, especially in rural settings with limited access to specialists.

## Background

Appendiceal neoplasms are rare, accounting for less than 1% of intestinal neoplasms [1]. There are three different types of appendiceal neoplasms, which are defined by the World Health Organization as mucinous adenoma, low-grade appendiceal mucinous neoplasm (LAMN), and mucinous appendiceal adenocarcinoma (MAA) [2]. Among primary malignant neoplasms of the appendix, MAA presents most frequently (37% of all appendiceal neoplasms) and its incidence in the United States is rising [3, 4]. Mucinous adenomas are characterized as benign masses confined to the mucosa with intact muscularis. LAMN and mucinous adenocarcinoma have the potential to cause pseudomyxoma peritonei (PMP) due to excessive mucin production. PMP is a spread of gelatinous material (mucin) from the lumen of the appendix into the abdominal cavity, morphologically referred to as mucoceles [5]. The median time to PMP development after MAA onset is ~ 2 years [6]. With peritoneal metastasis, the five-year survival rate for mucinous adenocarcinoma is reported as low as 25% [6]. In addition, mucinous adenocarcinomas have a relatively higher risk for hematogenous and nodal metastasis [6]. Therefore, the early detection of a mucinous appendiceal neoplasm is imperative.

Despite their clinical severity, the prospective clinical diagnosis of mucinous neoplasms may be difficult, as the symptoms are typically minimal or nonspecific. If symptoms do occur, mucinous neoplasms often present as abdominal pain, weight loss, nausea, vomiting, a palpable mass, and acute appendicitis. Additionally, the prevalence and clinical presentations may differ between sexes [5, 6]. Appendiceal mucoceles are more common in women (4:1) and therefore may be misdiagnosed as gynecological pathologies such as a cystic right adnexal mass of the ovary or fallopian tube [5, 6]. MAA may also be mistaken for a urinary tract infection or bladder cancer as it can present with hematuria in both men and women [7–16]. In rare cases, MAAs present as scrotal pain due to metastasis and may even been found within de Garengeot hernias [17, 18], which are femoral hernias containing the appendix.

Half of all mucinous neoplasms are diagnosed through incidental appendectomies or imaging studies [19]. By the time they are diagnosed, the neoplasms are often at an advanced stage [3]. Here, we report a rare patient presentation with late-stage MAA and review the common and unique clinical presentations with imaging findings to aid in future early diagnosis of MAA in undifferentiated patients.

## Case presentation

A 67-year-old male presented complaining of a right linear inguinal crease pustule that had persisted for 2 weeks. The pertinent vital signs were a temperature of 36.4 °C, and a weight of 91.6 kg. The patient was a current smoker with a 1/2 pack per day habit for the past 55 years. Past medical history was unremarkable with a history of right inguinal hernia repair and vasectomy. The patient was not taking any medications or over the counter supplements. His family history was unremarkable. The abdominal exam was remarkable for a small inguinal hernia and a 1-cm healed ulcer in the right inguinal area.

Four months later, the patient returned complaining of increased urinary frequency, hematuria, and scrotal pain lasting several days. There was no history of chills, nausea, vomiting, costovertebral angle (CVA) tenderness, or abdominal pain. His temperature was 38.0 °C. Urinalysis revealed + 1 blood, + 1 leukocyte, WBC > 182 HPF, RBC > 61 HPF, and positive for bacteria. Urine culture was positive for *Escherichia coli* and ciprofloxacin was subsequently prescribed. An ultrasound of the scrotum was not performed. The patient’s symptoms had resolved three weeks later at follow-up; however, the patient’s weight had decreased by 5.4 kg. A urinalysis again demonstrated 22 WBC/HPF and RBCs, and the follow-up culture was negative. Other labs were obtained, including CBC, CMP, and PSA, which were unremarkable.

**Fig. 1:**
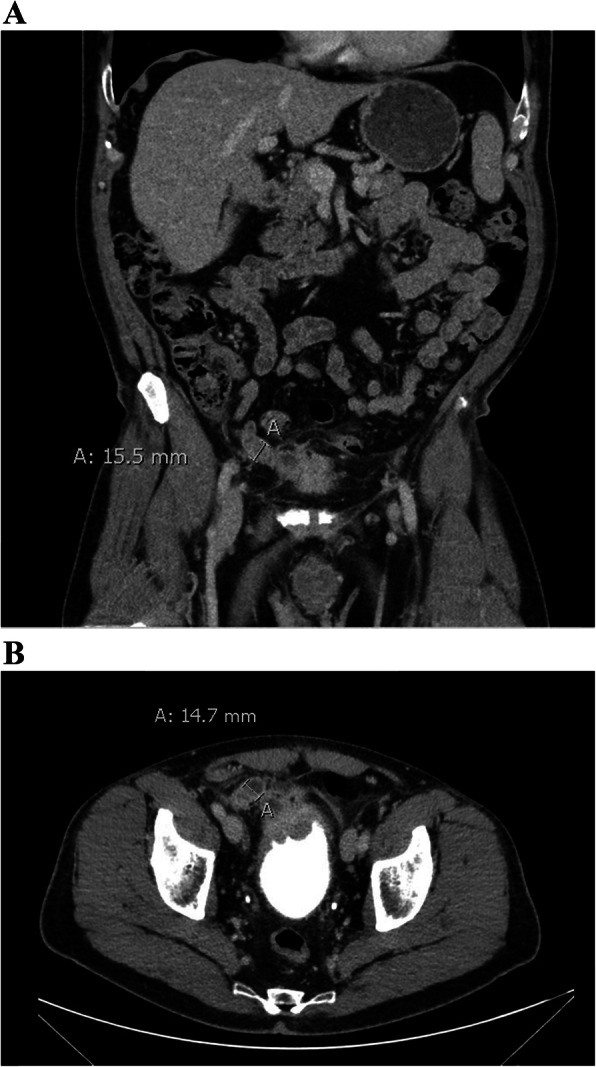
CT with contrast of abdomen and pelvis. **a**. CT with contrast of abdomen and pelvis (coronal plane). An estimated appendiceal luminal dilation of 15.5 mm (left) with fistula to the bladder is displayed. **b**. CT with contrast of abdomen and pelvis (axial plane). An estimated appendiceal luminal dilation of 14.7 mm (left), with fistula formation to the bladder is displayed

Two months later, the patient returned complaining of posterior scrotum abscess drainage. He was afebrile with no palpable testicular mass or tenderness. However, there was thickening and serosanguinous drainage over the right testicle. The patient was treated with trimethoprim/sulfamethoxazole and cephalexin and was referred to urology for his scrotal abscess. Later, computed tomography (CT) of the abdomen and pelvis with contrast revealed a distended appendix (> 1.3 cm) and a fistula located between the appendix, urinary bladder, right scrotum, and right groin (Fig. 1a and b). The patient was referred for colorectal surgery.

Subsequently, a shave biopsy of a scrotal abscess lesion revealed granulated tissue. Cystoscopy showed a large mass in the anterior right bladder wall. Transurethral resection of the bladder tumor (TURBT) revealed the presence of urothelial mucosa consistent with chronic inflammation. A bone scan did not reveal evidence of metastatic disease.

**Fig. 2:**
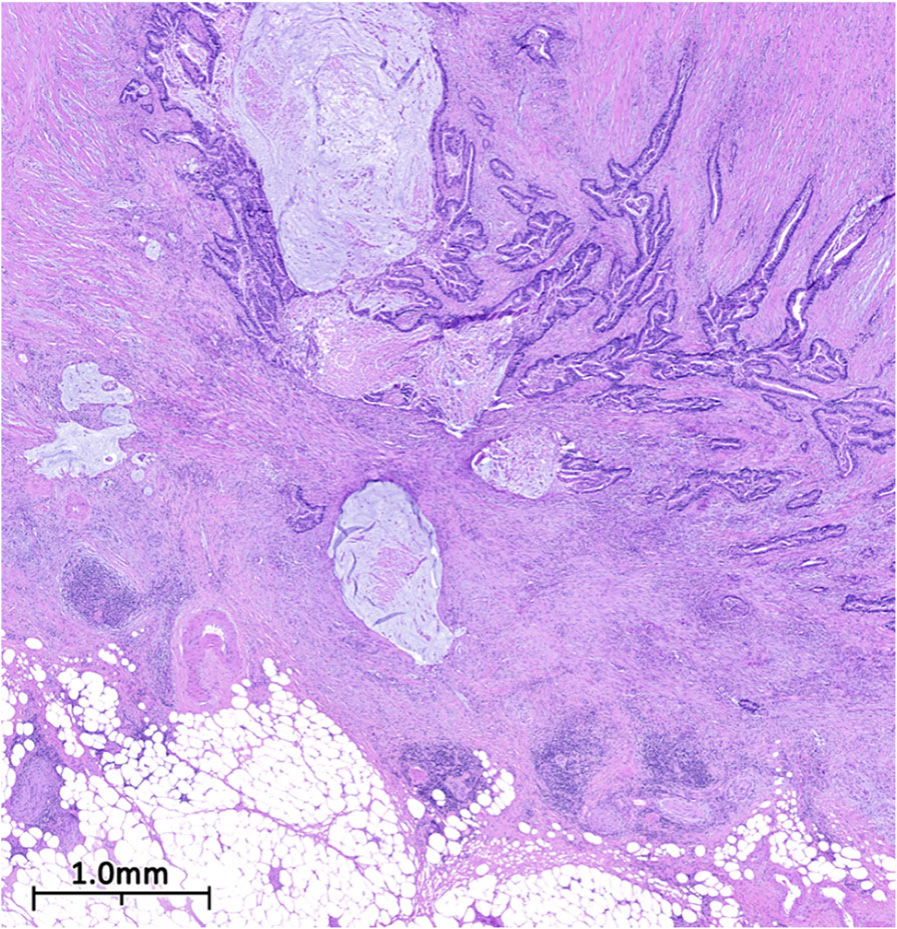
Low power photomicrograph of dissected appendiceal tissue. Figure 2. Low power photomicrograph of H&E stain showing moderately differentiated branching neoplastic glands focally distended by mucinous material and invading the muscularis. Mucinous pools partially lined with neoplastic epithelium are also present

**Fig. 3:**
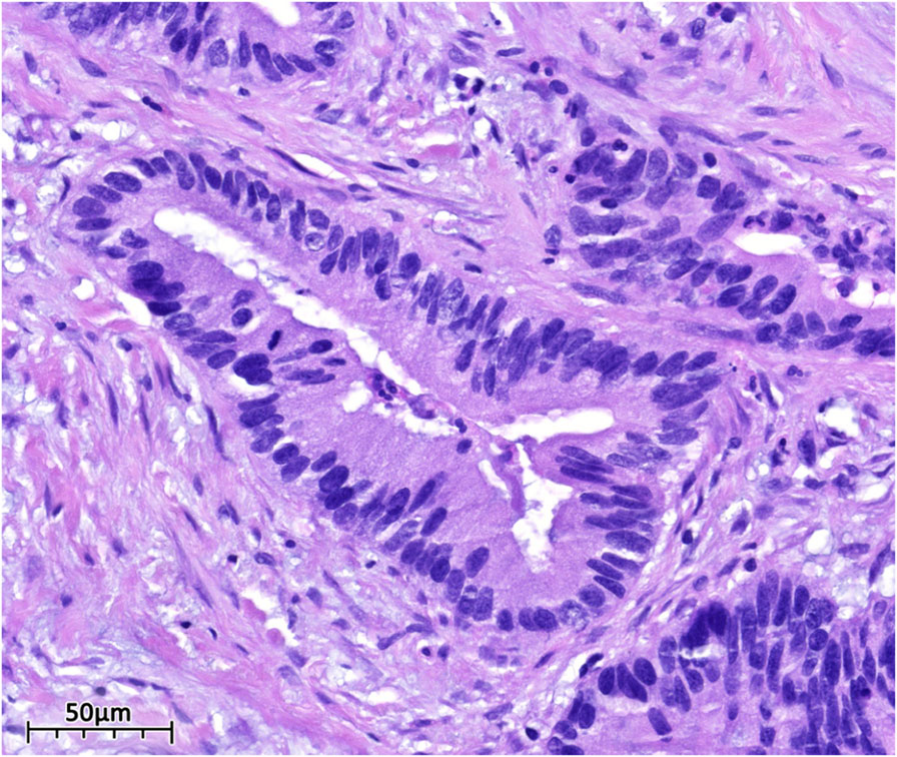
High power photomicrograph of dissected appendiceal tissue. Figure 3. High power photomicrograph of H&E stain showing moderately differentiated neoplastic glands lined by cells with nuclear pleomorphism and hyperchromasia (Hematoxylin and Eosin)

The patient then underwent a laparoscopic appendectomy, where an abundance of mucin was observed. This was followed by a right hemicolectomy, partial omentectomy, and lymph node dissection. Microscopic examination of the appendix revealed a mucinous adenocarcinoma that had invaded through the muscularis propria into the subserosa without lymphovascular invasion. The tumor was staged as pT3b pN0 M0 G2 (Figs. 2 and 3). After discharge, the patient was referred to oncology and FOLFOX regimen (levoleucovorin, fluorouracil, and oxaliplatin) was recommended.

## Discussion and conclusion

### Unique patient presentations

We describe a unique presentation of appendiceal MAA. To our knowledge, this is the first case study describing the diagnosis of late-stage appendiceal MAA presenting with scrotal pain, abscess, and inguinal drainage. CT imaging revealed appendix distension with > 1.3 cm luminal dilation (Figs. 1 and 2), which raises the strong suspicion for MAA [20]. Several case studies have reported right inguinal and right scrotal pain as presenting symptoms in a patient with acute appendicitis or a perforation of an appendiceal abscess [21–24].

### Image findings

Appendiceal mucoceles are frequently found using CT. Mucoceles can become inflamed and have a similar appearance on CT to acute appendicitis without mucocele. A CT cannot distinguish between benign (mucosal hyperplasia, retention cyst, mucinous adenoma) and malignant (LAMN, mucinous carcinoma) causes of appendiceal mucoceles [6]. However, few findings are specific for appendiceal neoplasms. The presence of appendiceal mural calcifications on CT is reported as 99.3% specific for appendiceal epithelial neoplasms [25]. Cystic dilation of the appendix, mural calcification, or maximum luminal dilation > 1.3 cm with CT have a 71% sensitivity, 94% specificity, and overall diagnostic accuracy of 88% for coexisting mucoceles with acute appendicitis [20].

Due to the increased incidence and frequent advanced stage diagnosis of mucinous appendiceal adenocarcinomas (MAA), the need of case reports highlighting unique features of this tumor type is imperative. This case highlights a late stage diagnosis and unique clinical features of MAA.

## Data Availability

Not applicable.

## Abbreviations

MAA: Mucinous appendiceal adenocarcinoma
PMP: Pseudomyxoma peritonei
TURBT: Transurethral resection of bladder tumor
CT: Computed tomography
LAMN: Low-grade appendiceal mucinous neoplasm
HIPEC: Heated intraperitoneal chemotherapy
H&E: Hematoxylin and Eosin
